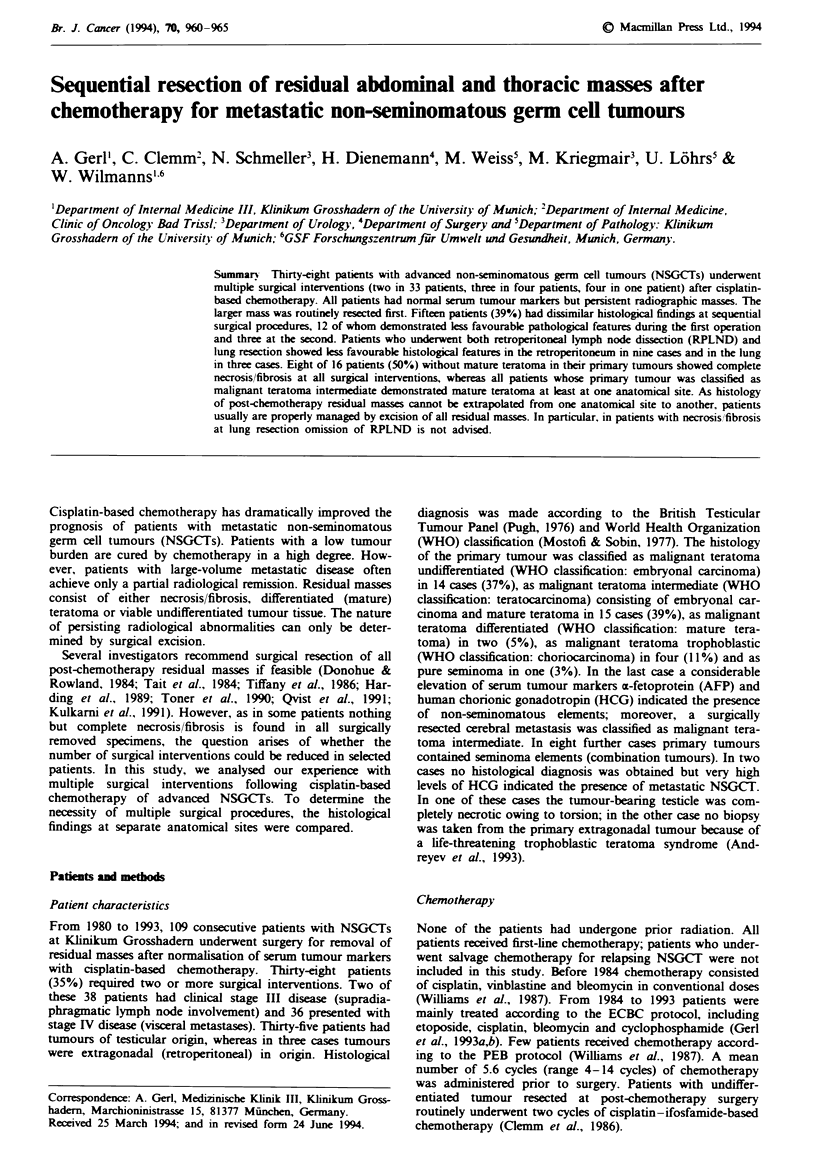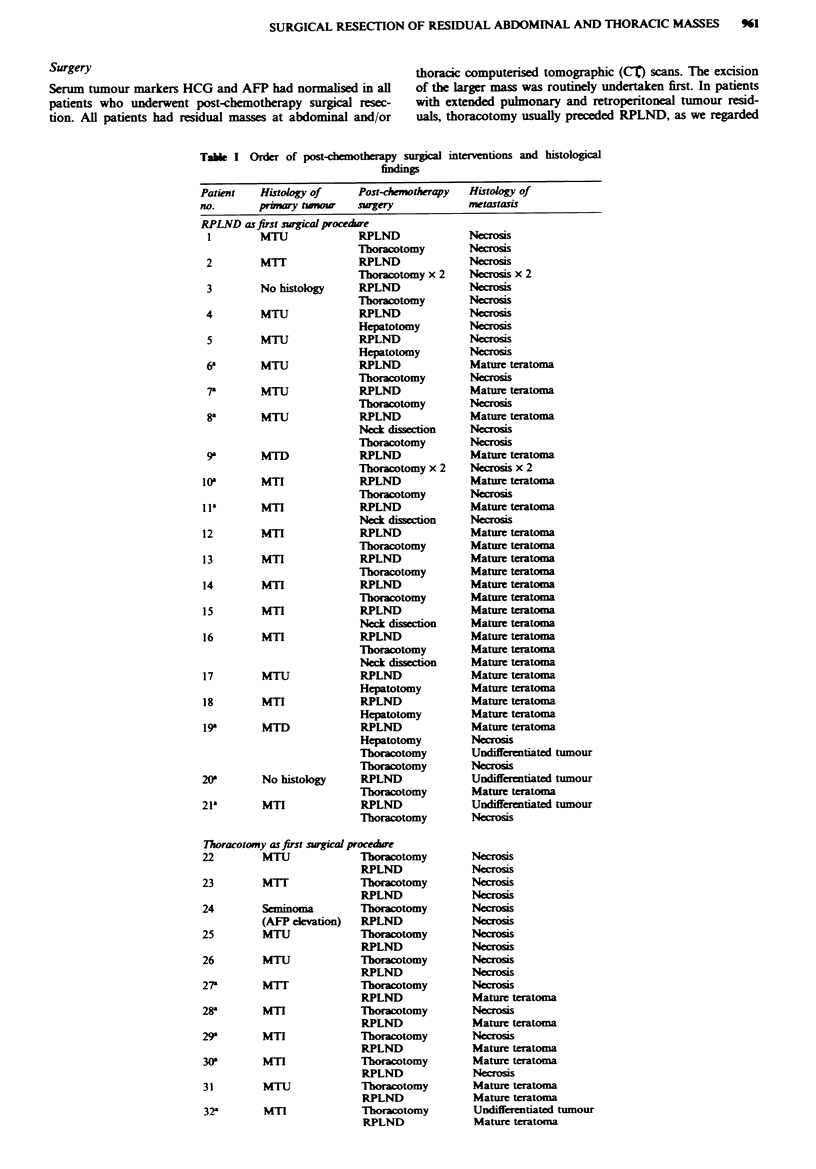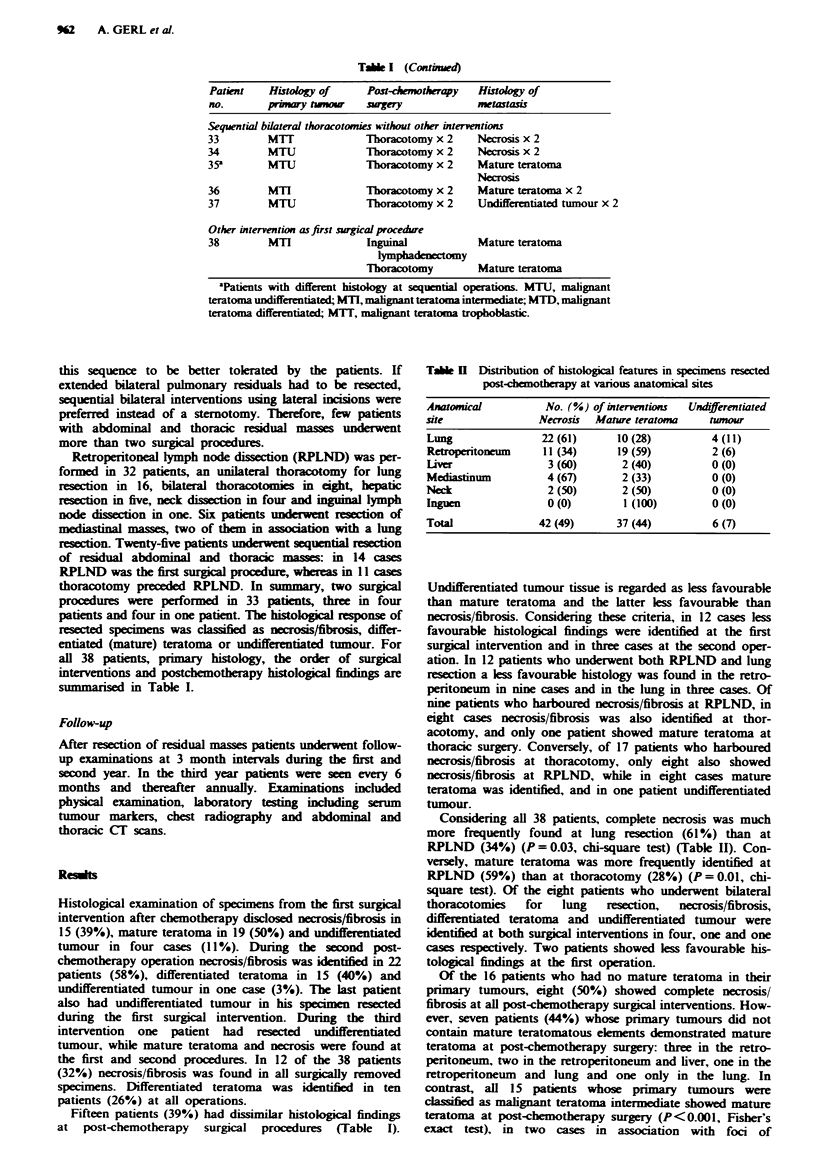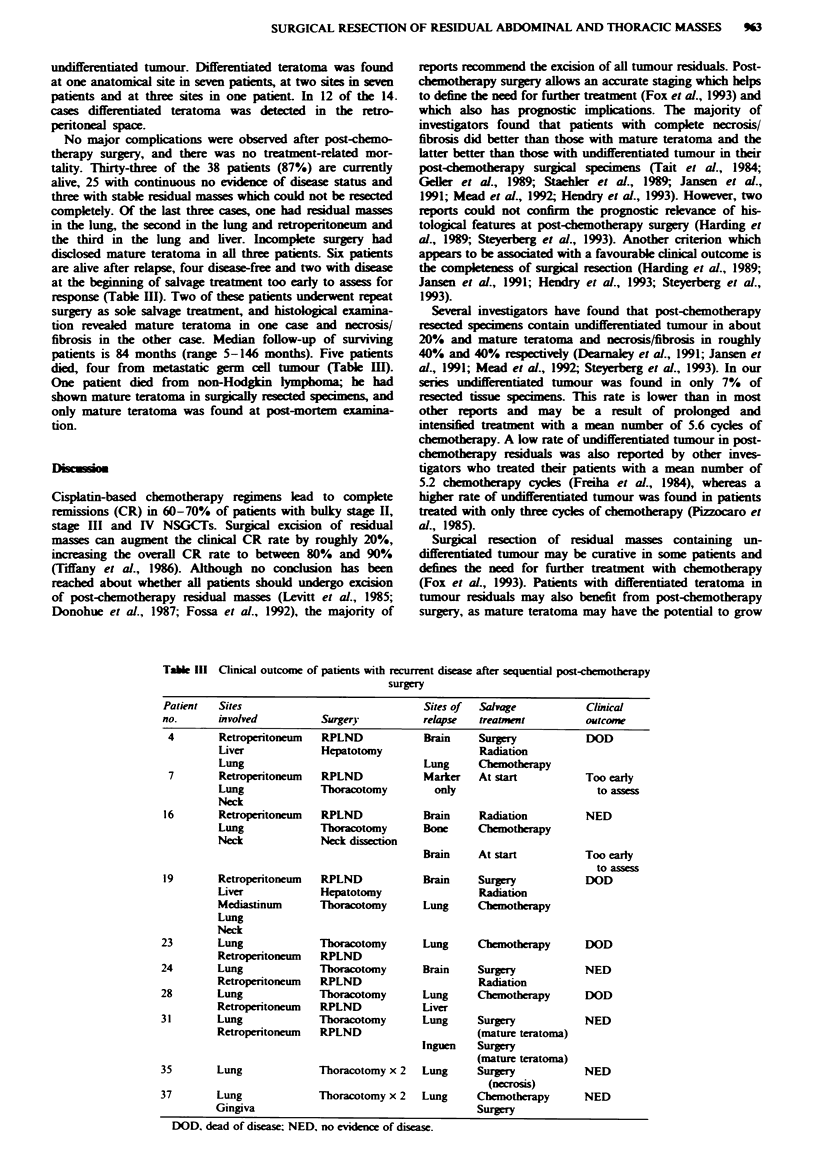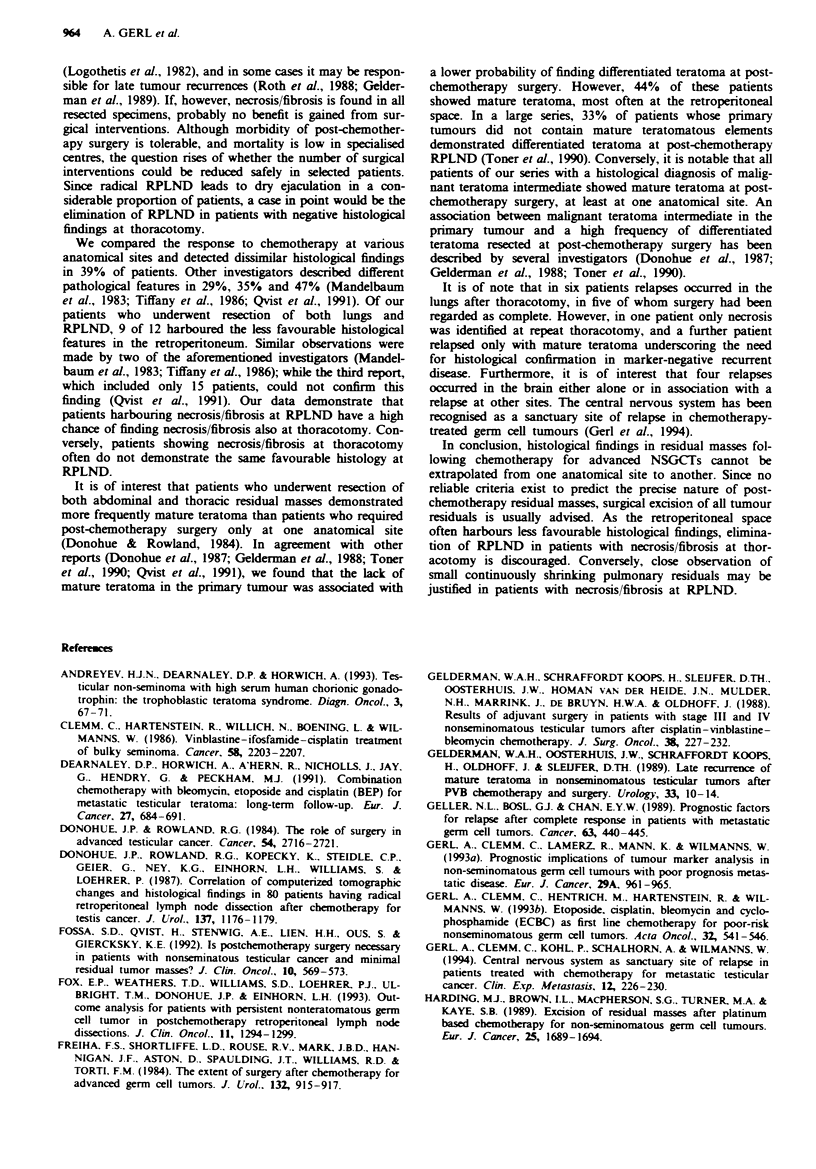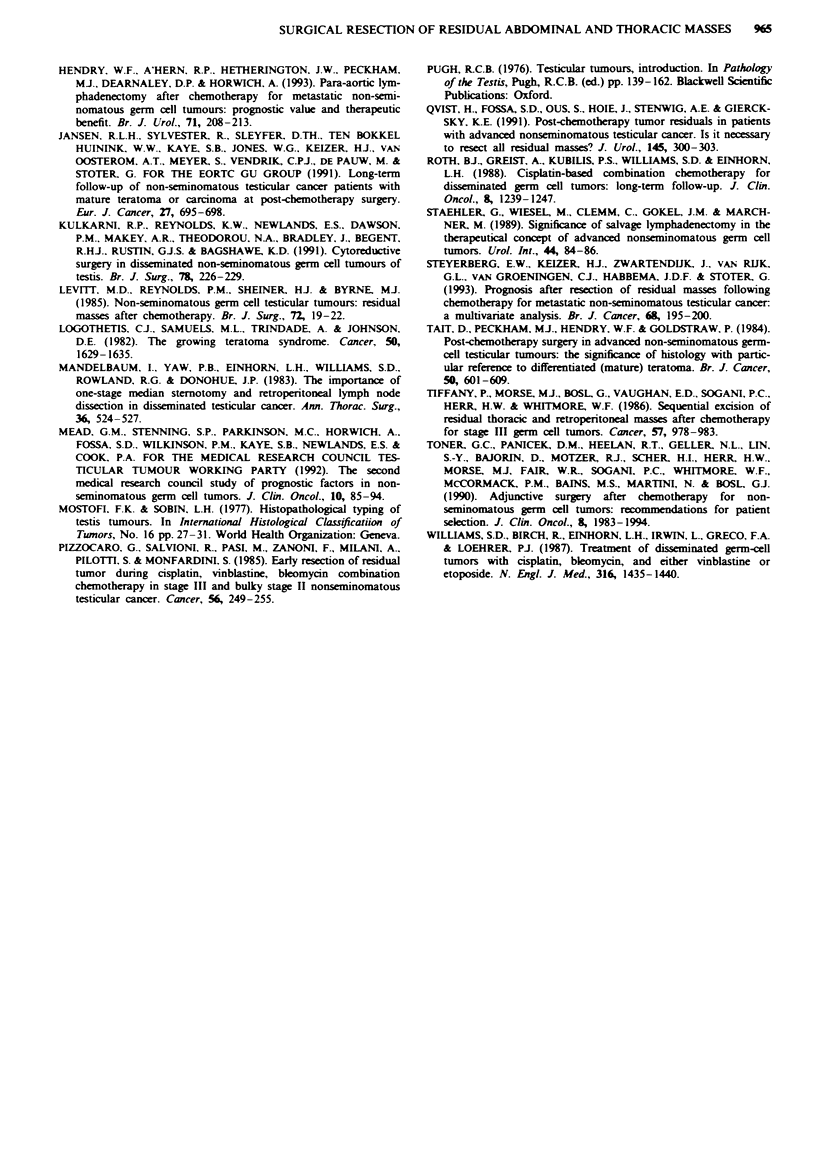# Sequential resection of residual abdominal and thoracic masses after chemotherapy for metastatic non-seminomatous germ cell tumours.

**DOI:** 10.1038/bjc.1994.429

**Published:** 1994-11

**Authors:** A. Gerl, C. Clemm, N. Schmeller, H. Dienemann, M. Weiss, M. Kriegmair, U. Löhrs, W. Wilmanns

**Affiliations:** Department of Internal Medicine III, Klinikum Grosshadern, University of Munich, Germany.

## Abstract

Thirty-eight patients with advanced non-seminomatous germ cell tumours (NSGCTs) underwent multiple surgical interventions (two in 33 patients, three in four patients, four in one patient) after cisplatin-based chemotherapy. All patients had normal serum tumour markers but persistent radiographic masses. The larger mass was routinely resected first. Fifteen patients (39%) had dissimilar histological findings at sequential surgical procedures, 12 of whom demonstrated less favourable pathological features during the first operation and three at the second. Patients who underwent both retroperitoneal lymph node dissection (RPLND) and lung resection showed less favourable histological features in the retroperitoneum in nine cases and in the lung in three cases. Eight of 16 patients (50%) without mature teratoma in their primary tumours showed complete necrosis/fibrosis at all surgical interventions, whereas all patients whose primary tumour was classified as malignant teratoma intermediate demonstrated mature teratoma at least at one anatomical site. As histology of post-chemotherapy residual masses cannot be extrapolated from one anatomical site to another, patients usually are properly managed by excision of all residual masses. In particular, in patients with necrosis/fibrosis at lung resection omission of RPLND is not advised.


Br. J. Cancer (1994), 70, 960-965 © Macmillan Press Ltd., 1994

### Sequential resection of residual abdominal and thoracic masses after chemotherapy for metastatic non-seminomatous germ cell tumours

A. Gerl[1], C. Clemm[2], N. Schmeller[3], H. Dienemann[4], M. Weiss[5], M. Kriegmair[3], U. Löhrs[5] & W. Wilmanns[1,6]

[1]*Department of Internal Medicine III, Klinikum Grosshadern of the University of Munich;* [2]*Department of Internal Medicine, Clinic of Oncology Bad Trissl;* [3]*Department of Urology,* [4]*Department of Surgery and* [5]*Department of Pathology: Klinikum Grosshadern of the University of Munich;* [6]*GSF Forschungszentrum für Umwelt und Gesundheit, Munich, Germany.*

**Summary** Thirty-eight patients with advanced non-seminomatous germ cell tumours (NSGCTs) underwent multiple surgical interventions (two in 33 patients, three in four patients, four in one patient) after cisplatin-based chemotherapy. All patients had normal serum tumour markers but persistent radiographic masses. The larger mass was routinely resected first. Fifteen patients (39%) had dissimilar histological findings at sequential surgical procedures, 12 of whom demonstrated less favourable pathological features during the first operation and three at the second. Patients who underwent both retroperitoneal lymph node dissection (RPLND) and lung resection showed less favourable histological features in the retroperitoneum in nine cases and in the lung in three cases. Eight of 16 patients (50%) without mature teratoma in their primary tumours showed complete necrosis/fibrosis at all surgical interventions, whereas all patients whose primary tumour was classified as malignant teratoma intermediate demonstrated mature teratoma at least at one anatomical site. As histology of post-chemotherapy residual masses cannot be extrapolated from one anatomical site to another, patients usually are properly managed by excision of all residual masses. In particular, in patients with necrosis/fibrosis at lung resection omission of RPLND is not advised.

Cisplatin-based chemotherapy has dramatically improved the prognosis of patients with metastatic non-seminomatous germ cell tumours (NSGCTs). Patients with a low tumour burden are cured by chemotherapy in a high degree. However, patients with large-volume metastatic disease often achieve only a partial radiological remission. Residual masses consist of either necrosis/fibrosis, differentiated (mature) teratoma or viable undifferentiated tumour tissue. The nature of persisting radiological abnormalities can only be determined by surgical excision.

Several investigators recommend surgical resection of all post-chemotherapy residual masses if feasible (Donohue & Rowland, 1984; Tait *et al.*, 1984; Tiffany *et al.*, 1986; Harding *et al.*, 1989; Toner *et al.*, 1990; Qvist *et al.*, 1991; Kulkarni *et al.*, 1991). However, as in some patients nothing but complete necrosis/fibrosis is found in all surgically removed specimens, the question arises of whether the number of surgical interventions could be reduced in selected patients. In this study, we analysed our experience with multiple surgical interventions following cisplatin-based chemotherapy of advanced NSGCTs. To determine the necessity of multiple surgical procedures, the histological findings at separate anatomical sites were compared.

### Patients and methods

### *Patient characteristics*

From 1980 to 1993, 109 consecutive patients with NSGCTs at Klinikum Grosshadern underwent surgery for removal of residual masses after normalisation of serum tumour markers with cisplatin-based chemotherapy. Thirty-eight patients (35%) required two or more surgical interventions. Two of these 38 patients had clinical stage III disease (supradiaphragmatic lymph node involvement) and 36 presented with stage IV disease (visceral metastases). Thirty-five patients had tumours of testicular origin, whereas in three cases tumours were extragonadal (retroperitoneal) in origin. Histological diagnosis was made according to the British Testicular Tumour Panel (Pugh, 1976) and World Health Organization (WHO) classification (Mostofi & Sobin, 1977). The histology of the primary tumour was classified as malignant teratoma undifferentiated (WHO classification: embryonal carcinoma) in 14 cases (37%), as malignant teratoma intermediate (WHO classification: teratocarcinoma) consisting of embryonal carcinoma and mature teratoma in 15 cases (39%), as malignant teratoma differentiated (WHO classification: mature teratoma) in two (5%), as malignant teratoma trophoblastic (WHO classification: choriocarcinoma) in four (11%) and as pure seminoma in one (3%). In the last case a considerable elevation of serum tumour markers α-fetoprotein (AFP) and human chorionic gonadotropin (HCG) indicated the presence of non-seminomatous elements; moreover, a surgically resected cerebral metastasis was classified as malignant teratoma intermediate. In eight further cases primary tumours contained seminoma elements (combination tumours). In two cases no histological diagnosis was obtained but very high levels of HCG indicated the presence of metastatic NSGCT. In one of these cases the tumour-bearing testicle was completely necrotic owing to torsion; in the other case no biopsy was taken from the primary extragonadal tumour because of a life-threatening trophoblastic teratoma syndrome (Andreyev *et al.*, 1993).

### *Chemotherapy*

None of the patients had undergone prior radiation. All patients received first-line chemotherapy; patients who underwent salvage chemotherapy for relapsing NSGCT were not included in this study. Before 1984 chemotherapy consisted of cisplatin, vinblastine and bleomycin in conventional doses (Williams *et al.*, 1987). From 1984 to 1993 patients were mainly treated according to the ECBC protocol, including etoposide, cisplatin, bleomycin and cyclophosphamide (Gerl *et al.*, 1993*a*,*b*). Few patients received chemotherapy according to the PEB protocol (Williams *et al.*, 1987). A mean number of 5.6 cycles (range 4–14 cycles) of chemotherapy was administered prior to surgery. Patients with undifferentiated tumour resected at post-chemotherapy surgery routinely underwent two cycles of cisplatin–ifosfamide-based chemotherapy (Clemm *et al.*, 1986).

Correspondence: A. Gerl, Medizinische Klinik III, Klinikum Grosshadern, Marchioninistrasse 15, 81377 München, Germany.
Received 25 March 1994; and in revised form 24 June 1994.

*Surgery*

Serum tumour markers HCG and AFP had normalised in all patients who underwent post-chemotherapy surgical resection. All patients had residual masses at abdominal and/or thoracic computerised tomographic (CT) scans. The excision of the larger mass was routinely undertaken first. In patients with extended pulmonary and retroperitoneal tumour residuals, thoracotomy usually preceded RPLND, as we regarded

**Table I** Order of post-chemotherapy surgical interventions and histological findings

| *Patient no.* | *Histology of primary tumour* | *Post-chemotherapy surgery* | *Histology of metastasis* |
|---|---|---|---|
| *RPLND as first surgical procedure* | | | |
| 1 | MTU | RPLND | Necrosis |
| | | Thoracotomy | Necrosis |
| 2 | MTT | RPLND | Necrosis |
| | | Thoracotomy × 2 | Necrosis × 2 |
| 3 | No histology | RPLND | Necrosis |
| | | Thoracotomy | Necrosis |
| 4 | MTU | RPLND | Necrosis |
| | | Hepatotomy | Necrosis |
| 5 | MTU | RPLND | Necrosis |
| | | Hepatotomy | Necrosis |
| 6[a] | MTU | RPLND | Mature teratoma |
| | | Thoracotomy | Necrosis |
| 7[a] | MTU | RPLND | Mature teratoma |
| | | Thoracotomy | Necrosis |
| 8[a] | MTU | RPLND | Mature teratoma |
| | | Neck dissection | Necrosis |
| | | Thoracotomy | Necrosis |
| 9[a] | MTD | RPLND | Mature teratoma |
| | | Thoracotomy × 2 | Necrosis × 2 |
| 10[a] | MTI | RPLND | Mature teratoma |
| | | Thoracotomy | Necrosis |
| 11[a] | MTI | RPLND | Mature teratoma |
| | | Neck dissection | Necrosis |
| 12 | MTI | RPLND | Mature teratoma |
| | | Thoracotomy | Mature teratoma |
| 13 | MTI | RPLND | Mature teratoma |
| | | Thoracotomy | Mature teratoma |
| 14 | MTI | RPLND | Mature teratoma |
| | | Thoracotomy | Mature teratoma |
| 15 | MTI | RPLND | Mature teratoma |
| | | Neck dissection | Mature teratoma |
| 16 | MTI | RPLND | Mature teratoma |
| | | Thoracotomy | Mature teratoma |
| | | Neck dissection | Mature teratoma |
| 17 | MTU | RPLND | Mature teratoma |
| | | Hepatotomy | Mature teratoma |
| 18 | MTI | RPLND | Mature teratoma |
| | | Hepatotomy | Mature teratoma |
| 19[a] | MTD | RPLND | Mature teratoma |
| | | Hepatotomy | Necrosis |
| | | Thoracotomy | Undifferentiated tumour |
| | | Thoracotomy | Necrosis |
| 20[a] | No histology | RPLND | Undifferentiated tumour |
| | | Thoracotomy | Mature teratoma |
| 21[a] | MTI | RPLND | Undifferentiated tumour |
| | | Thoracotomy | Necrosis |
| *Thoracotomy as first surgical procedure* | | | |
| 22 | MTU | Thoracotomy | Necrosis |
| | | RPLND | Necrosis |
| 23 | MTT | Thoracotomy | Necrosis |
| | | RPLND | Necrosis |
| 24 | Seminoma | Thoracotomy | Necrosis |
| | (AFP elevation) | RPLND | Necrosis |
| 25 | MTU | Thoracotomy | Necrosis |
| | | RPLND | Necrosis |
| 26 | MTU | Thoracotomy | Necrosis |
| | | RPLND | Necrosis |
| 27[a] | MTT | Thoracotomy | Necrosis |
| | | RPLND | Mature teratoma |
| 28[a] | MTI | Thoracotomy | Necrosis |
| | | RPLND | Mature teratoma |
| 29[a] | MTI | Thoracotomy | Necrosis |
| | | RPLND | Mature teratoma |
| 30[a] | MTI | Thoracotomy | Mature teratoma |
| | | RPLND | Necrosis |
| 31 | MTU | Thoracotomy | Mature teratoma |
| | | RPLND | Mature teratoma |
| 32[a] | MTI | Thoracotomy | Undifferentiated tumour |
| | | RPLND | Mature teratoma |

**Table I** (*Continued*)

| *Patient no.* | *Histology of primary tumour* | *Post-chemotherapy surgery* | *Histology of metastasis* |
|---|---|---|---|
| *Sequential bilateral thoracotomies without other interventions* | | | |
| 33 | MTT | Thoracotomy x 2 | Necrosis x 2 |
| 34 | MTU | Thoracotomy x 2 | Necrosis x 2 |
| 35[a] | MTU | Thoracotomy x 2 | Mature teratoma<br>Necrosis |
| 36 | MTI | Thoracotomy x 2 | Mature teratoma x 2 |
| 37 | MTU | Thoracotomy x 2 | Undifferentiated tumour x 2 |
| *Other intervention as first surgical procedure* | | | |
| 38 | MTI | Inguinal lymphadenectomy | Mature teratoma |
| | | Thoracotomy | Mature teratoma |

[a]Patients with different histology at sequential operations. MTU, malignant teratoma undifferentiated; MTI, malignant teratoma intermediate; MTD, malignant teratoma differentiated; MTT, malignant teratoma trophoblastic.

this sequence to be better tolerated by the patients. If extended bilateral pulmonary residuals had to be resected, sequential bilateral interventions using lateral incisions were preferred instead of a sternotomy. Therefore, few patients with abdominal and thoracic residual masses underwent more than two surgical procedures.

Retroperitoneal lymph node dissection (RPLND) was performed in 32 patients, an unilateral thoracotomy for lung resection in 16, bilateral thoracotomies in eight, hepatic resection in five, neck dissection in four and inguinal lymph node dissection in one. Six patients underwent resection of mediastinal masses, two of them in association with a lung resection. Twenty-five patients underwent sequential resection of residual abdominal and thoracic masses: in 14 cases RPLND was the first surgical procedure, whereas in 11 cases thoracotomy preceded RPLND. In summary, two surgical procedures were performed in 33 patients, three in four patients and four in one patient. The histological response of resected specimens was classified as necrosis/fibrosis, differentiated (mature) teratoma or undifferentiated tumour. For all 38 patients, primary histology, the order of surgical interventions and postchemotherapy histological findings are summarised in Table I.

### *Follow-up*

After resection of residual masses patients underwent follow-up examinations at 3 month intervals during the first and second year. In the third year patients were seen every 6 months and thereafter annually. Examinations included physical examination, laboratory testing including serum tumour markers, chest radiography and abdominal and thoracic CT scans.

### Results

Histological examination of specimens from the first surgical intervention after chemotherapy disclosed necrosis/fibrosis in 15 (39%), mature teratoma in 19 (50%) and undifferentiated tumour in four cases (11%). During the second post-chemotherapy operation necrosis/fibrosis was identified in 22 patients (58%), differentiated teratoma in 15 (40%) and undifferentiated tumour in one case (3%). The last patient also had undifferentiated tumour in his specimen resected during the first surgical intervention. During the third intervention one patient had resected undifferentiated tumour, while mature teratoma and necrosis were found at the first and second procedures. In 12 of the 38 patients (32%) necrosis/fibrosis was found in all surgically removed specimens. Differentiated teratoma was identified in ten patients (26%) at all operations.

Fifteen patients (39%) had dissimilar histological findings at post-chemotherapy surgical procedures (Table I).

**Table II** Distribution of histological features in specimens resected post-chemotherapy at various anatomical sites

| *Anatomical site* | *No. (%) of interventions* *Necrosis* | *Mature teratoma* | *Undifferentiated tumour* |
|---|---|---|---|
| Lung | 22 (61) | 10 (28) | 4 (11) |
| Retroperitoneum | 11 (34) | 19 (59) | 2 (6) |
| Liver | 3 (60) | 2 (40) | 0 (0) |
| Mediastinum | 4 (67) | 2 (33) | 0 (0) |
| Neck | 2 (50) | 2 (50) | 0 (0) |
| Inguen | 0 (0) | 1 (100) | 0 (0) |
| Total | 42 (49) | 37 (44) | 6 (7) |

Undifferentiated tumour tissue is regarded as less favourable than mature teratoma and the latter less favourable than necrosis/fibrosis. Considering these criteria, in 12 cases less favourable histological findings were identified at the first surgical intervention and in three cases at the second operation. In 12 patients who underwent both RPLND and lung resection a less favourable histology was found in the retroperitoneum in nine cases and in the lung in three cases. Of nine patients who harboured necrosis/fibrosis at RPLND, in eight cases necrosis/fibrosis was also identified at thoracotomy, and only one patient showed mature teratoma at thoracic surgery. Conversely, of 17 patients who harboured necrosis/fibrosis at thoracotomy, only eight also showed necrosis/fibrosis at RPLND, while in eight cases mature teratoma was identified, and in one patient undifferentiated tumour.

Considering all 38 patients, complete necrosis was much more frequently found at lung resection (61%) than at RPLND (34%) ($P = 0.03$, chi-square test) (Table II). Conversely, mature teratoma was more frequently identified at RPLND (59%) than at thoracotomy (28%) ($P = 0.01$, chi-square test). Of the eight patients who underwent bilateral thoracotomies for lung resection, necrosis/fibrosis, differentiated teratoma and undifferentiated tumour were identified at both surgical interventions in four, one and one cases respectively. Two patients showed less favourable histological findings at the first operation.

Of the 16 patients who had no mature teratoma in their primary tumours, eight (50%) showed complete necrosis/fibrosis at all post-chemotherapy surgical interventions. However, seven patients (44%) whose primary tumours did not contain mature teratomatous elements demonstrated mature teratoma at post-chemotherapy surgery: three in the retroperitoneum, two in the retroperitoneum and liver, one in the retroperitoneum and lung and one only in the lung. In contrast, all 15 patients whose primary tumours were classified as malignant teratoma intermediate showed mature teratoma at post-chemotherapy surgery ($P < 0.001$, Fisher's exact test), in two cases in association with foci of undifferentiated tumour. Differentiated teratoma was found at one anatomical site in seven patients, at two sites in seven patients and at three sites in one patient. In 12 of the 14 cases differentiated teratoma was detected in the retroperitoneal space.

No major complications were observed after post-chemotherapy surgery, and there was no treatment-related mortality. Thirty-three of the 38 patients (87%) are currently alive, 25 with continuous no evidence of disease status and three with stable residual masses which could not be resected completely. Of the last three cases, one had residual masses in the lung, the second in the lung and retroperitoneum and the third in the lung and liver. Incomplete surgery had disclosed mature teratoma in all three patients. Six patients are alive after relapse, four disease-free and two with disease at the beginning of salvage treatment too early to assess for response (Table III). Two of these patients underwent repeat surgery as sole salvage treatment, and histological examination revealed mature teratoma in one case and necrosis/fibrosis in the other case. Median follow-up of surviving patients is 84 months (range 5–146 months). Five patients died, four from metastatic germ cell tumour (Table III). One patient died from non-Hodgkin lymphoma; he had shown mature teratoma in surgically resected specimens, and only mature teratoma was found at post-mortem examination.

### Discussion

Cisplatin-based chemotherapy regimens lead to complete remissions (CR) in 60–70% of patients with bulky stage II, stage III and IV NSGCTs. Surgical excision of residual masses can augment the clinical CR rate by roughly 20%, increasing the overall CR rate to between 80% and 90% (Tiffany *et al.*, 1986). Although no conclusion has been reached about whether all patients should undergo excision of post-chemotherapy residual masses (Levitt *et al.*, 1985; Donohue *et al.*, 1987; Fossa *et al.*, 1992), the majority of reports recommend the excision of all tumour residuals. Post-chemotherapy surgery allows an accurate staging which helps to define the need for further treatment (Fox *et al.*, 1993) and which also has prognostic implications. The majority of investigators found that patients with complete necrosis/fibrosis did better than those with mature teratoma and the latter better than those with undifferentiated tumour in their post-chemotherapy surgical specimens (Tait *et al.*, 1984; Geller *et al.*, 1989; Staehler *et al.*, 1989; Jansen *et al.*, 1991; Mead *et al.*, 1992; Hendry *et al.*, 1993). However, two reports could not confirm the prognostic relevance of histological features at post-chemotherapy surgery (Harding *et al.*, 1989; Steyerberg *et al.*, 1993). Another criterion which appears to be associated with a favourable clinical outcome is the completeness of surgical resection (Harding *et al.*, 1989; Jansen *et al.*, 1991; Hendry *et al.*, 1993; Steyerberg *et al.*, 1993).

Several investigators have found that post-chemotherapy resected specimens contain undifferentiated tumour in about 20% and mature teratoma and necrosis/fibrosis in roughly 40% and 40% respectively (Dearnaley *et al.*, 1991; Jansen *et al.*, 1991; Mead *et al.*, 1992; Steyerberg *et al.*, 1993). In our series undifferentiated tumour was found in only 7% of resected tissue specimens. This rate is lower than in most other reports and may be a result of prolonged and intensified treatment with a mean number of 5.6 cycles of chemotherapy. A low rate of undifferentiated tumour in post-chemotherapy residuals was also reported by other investigators who treated their patients with a mean number of 5.2 chemotherapy cycles (Freiha *et al.*, 1984), whereas a higher rate of undifferentiated tumour was found in patients treated with only three cycles of chemotherapy (Pizzocaro *et al.*, 1985).

Surgical resection of residual masses containing undifferentiated tumour may be curative in some patients and defines the need for further treatment with chemotherapy (Fox *et al.*, 1993). Patients with differentiated teratoma in tumour residuals may also benefit from post-chemotherapy surgery, as mature teratoma may have the potential to grow

**Table III** Clinical outcome of patients with recurrent disease after sequential post-chemotherapy surgery

| *Patient no.* | *Sites involved* | *Surgery* | *Sites of relapse* | *Salvage treatment* | *Clinical outcome* |
|---|---|---|---|---|---|
| 4 | Retroperitoneum | RPLND | Brain | Surgery | DOD |
| | Liver | Hepatotomy | | Radiation | |
| | Lung | | Lung | Chemotherapy | |
| 7 | Retroperitoneum | RPLND | Marker only | At start | Too early to assess |
| | Lung | Thoracotomy | | | |
| | Neck | | | | |
| 16 | Retroperitoneum | RPLND | Brain | Radiation | NED |
| | Lung | Thoracotomy | Bone | Chemotherapy | |
| | Neck | Neck dissection | | | |
| | | | Brain | At start | Too early to assess |
| 19 | Retroperitoneum | RPLND | Brain | Surgery | DOD |
| | Liver | Hepatotomy | | Radiation | |
| | Mediastinum | Thoracotomy | Lung | Chemotherapy | |
| | Lung | | | | |
| | Neck | | | | |
| 23 | Lung | Thoracotomy | Lung | Chemotherapy | DOD |
| | Retroperitoneum | RPLND | | | |
| 24 | Lung | Thoracotomy | Brain | Surgery | NED |
| | Retroperitoneum | RPLND | | Radiation | |
| 28 | Lung | Thoracotomy | Lung | Chemotherapy | DOD |
| | Retroperitoneum | RPLND | Liver | | |
| 31 | Lung | Thoracotomy | Lung | Surgery (mature teratoma) | NED |
| | Retroperitoneum | RPLND | | | |
| | | | Inguen | Surgery (mature teratoma) | |
| 35 | Lung | Thoracotomy × 2 | Lung | Surgery (necrosis) | NED |
| 37 | Lung | Thoracotomy × 2 | Lung | Chemotherapy | NED |
| | Gingiva | | | Surgery | |

DOD, dead of disease; NED, no evidence of disease.

(Logothetis et al., 1982), and in some cases it may be responsible for late tumour recurrences (Roth et al., 1988; Gelderman et al., 1989). If, however, necrosis/fibrosis is found in all resected specimens, probably no benefit is gained from surgical interventions. Although morbidity of post-chemotherapy surgery is tolerable, and mortality is low in specialised centres, the question rises of whether the number of surgical interventions could be reduced safely in selected patients. Since radical RPLND leads to dry ejaculation in a considerable proportion of patients, a case in point would be the elimination of RPLND in patients with negative histological findings at thoracotomy.

We compared the response to chemotherapy at various anatomical sites and detected dissimilar histological findings in 39% of patients. Other investigators described different pathological features in 29%, 35% and 47% (Mandelbaum et al., 1983; Tiffany et al., 1986; Qvist et al., 1991). Of our patients who underwent resection of both lungs and RPLND, 9 of 12 harboured the less favourable histological features in the retroperitoneum. Similar observations were made by two of the aforementioned investigators (Mandelbaum et al., 1983; Tiffany et al., 1986); while the third report, which included only 15 patients, could not confirm this finding (Qvist et al., 1991). Our data demonstrate that patients harbouring necrosis/fibrosis at RPLND have a high chance of finding necrosis/fibrosis also at thoracotomy. Conversely, patients showing necrosis/fibrosis at thoracotomy often do not demonstrate the same favourable histology at RPLND.

It is of interest that patients who underwent resection of both abdominal and thoracic residual masses demonstrated more frequently mature teratoma than patients who required post-chemotherapy surgery only at one anatomical site (Donohue & Rowland, 1984). In agreement with other reports (Donohue et al., 1987; Gelderman et al., 1988; Toner et al., 1990; Qvist et al., 1991), we found that the lack of mature teratoma in the primary tumour was associated with a lower probability of finding differentiated teratoma at post-chemotherapy surgery. However, 44% of these patients showed mature teratoma, most often at the retroperitoneal space. In a large series, 33% of patients whose primary tumours did not contain mature teratomatous elements demonstrated differentiated teratoma at post-chemotherapy RPLND (Toner et al., 1990). Conversely, it is notable that all patients of our series with a histological diagnosis of malignant teratoma intermediate showed mature teratoma at post-chemotherapy surgery, at least at one anatomical site. An association between malignant teratoma intermediate in the primary tumour and a high frequency of differentiated teratoma resected at post-chemotherapy surgery has been described by several investigators (Donohue et al., 1987; Gelderman et al., 1988; Toner et al., 1990).

It is of note that in six patients relapses occurred in the lungs after thoracotomy, in five of whom surgery had been regarded as complete. However, in one patient only necrosis was identified at repeat thoracotomy, and a further patient relapsed only with mature teratoma underscoring the need for histological confirmation in marker-negative recurrent disease. Furthermore, it is of interest that four relapses occurred in the brain either alone or in association with a relapse at other sites. The central nervous system has been recognised as a sanctuary site of relapse in chemotherapy-treated germ cell tumours (Gerl et al., 1994).

In conclusion, histological findings in residual masses following chemotherapy for advanced NSGCTs cannot be extrapolated from one anatomical site to another. Since no reliable criteria exist to predict the precise nature of post-chemotherapy residual masses, surgical excision of all tumour residuals is usually advised. As the retroperitoneal space often harbours less favourable histological findings, elimination of RPLND in patients with necrosis/fibrosis at thoracotomy is discouraged. Conversely, close observation of small continuously shrinking pulmonary residuals may be justified in patients with necrosis/fibrosis at RPLND.

### References

ANDREYEV, H.J.N., DEARNALEY, D.P. & HORWICH, A. (1993). Testicular non-seminoma with high serum human chorionic gonadotrophin: the trophoblastic teratoma syndrome. Diagn. Oncol., 3, 67-71.

CLEMM, C., HARTENSTEIN, R., WILLICH, N., BOENING, L. & WILMANNS, W. (1986). Vinblastine-ifosfamide-cisplatin treatment of bulky seminoma. Cancer, 58, 2203-2207.

DEARNALEY, D.P., HORWICH, A., A'HERN, R., NICHOLLS, J., JAY, G., HENDRY, G. & PECKHAM, M.J. (1991). Combination chemotherapy with bleomycin, etoposide and cisplatin (BEP) for metastatic testicular teratoma: long-term follow-up. Eur. J. Cancer, 27, 684-691.

DONOHUE, J.P. & ROWLAND, R.G. (1984). The role of surgery in advanced testicular cancer. Cancer, 54, 2716-2721.

DONOHUE, J.P., ROWLAND, R.G., KOPECKY, K., STEIDLE, C.P., GEIER, G., NEY, K.G., EINHORN, L.H., WILLIAMS, S. & LOEHRER, P. (1987). Correlation of computerized tomographic changes and histological findings in 80 patients having radical retroperitoneal lymph node dissection after chemotherapy for testis cancer. J. Urol., 137, 1176-1179.

FOSSA, S.D., QVIST, H., STENWIG, A.E., LIEN, H.H., OUS, S. & GIERCKSKY, K.E. (1992). Is postchemotherapy surgery necessary in patients with nonseminatous testicular cancer and minimal residual tumor masses? J. Clin. Oncol., 10, 569-573.

FOX, E.P., WEATHERS, T.D., WILLIAMS, S.D., LOEHRER, P.J., ULBRIGHT, T.M., DONOHUE, J.P. & EINHORN, L.H. (1993). Outcome analysis for patients with persistent nonteratomatous germ cell tumor in postchemotherapy retroperitoneal lymph node dissections. J. Clin. Oncol., 11, 1294-1299.

FREIHA, F.S., SHORTLIFFE, L.D., ROUSE, R.V., MARK, J.B.D., HANNIGAN, J.F., ASTON, D., SPAULDING, J.T., WILLIAMS, R.D. & TORTI, F.M. (1984). The extent of surgery after chemotherapy for advanced germ cell tumors. J. Urol., 132, 915-917.

GELDERMAN, W.A.H., SCHRAFFORDT KOOPS, H., SLEIJFER, D.TH., OOSTERHUIS, J.W., HOMAN VAN DER HEIDE, J.N., MULDER, N.H., MARRINK, J., DE BRUYN, H.W.A. & OLDHOFF, J. (1988). Results of adjuvant surgery in patients with stage III and IV nonseminomatous testicular tumors after cisplatin-vinblastine-bleomycin chemotherapy. J. Surg. Oncol., 38, 227-232.

GELDERMAN, W.A.H., OOSTERHUIS, J.W., SCHRAFFORDT KOOPS, H., OLDHOFF, J. & SLEIJFER, D.TH. (1989). Late recurrence of mature teratoma in nonseminomatous testicular tumors after PVB chemotherapy and surgery. Urology, 33, 10-14.

GELLER, N.L., BOSL, G.J. & CHAN, E.Y.W. (1989). Prognostic factors for relapse after complete response in patients with metastatic germ cell tumors. Cancer, 63, 440-445.

GERL, A., CLEMM, C., LAMERZ, R., MANN, K. & WILMANNS, W. (1993a). Prognostic implications of tumour marker analysis in non-seminomatous germ cell tumours with poor prognosis metastatic disease. Eur. J. Cancer, 29A, 961-965.

GERL, A., CLEMM, C., HENTRICH, M., HARTENSTEIN, R. & WILMANNS, W. (1993b). Etoposide, cisplatin, bleomycin and cyclophosphamide (ECBC) as first line chemotherapy for poor-risk nonseminomatous germ cell tumors. Acta Oncol., 32, 541-546.

GERL, A., CLEMM, C., KOHL, P., SCHALHORN, A. & WILMANNS, W. (1994). Central nervous system as sanctuary site of relapse in patients treated with chemotherapy for metastatic testicular cancer. Clin. Exp. Metastasis, 12, 226-230.

HARDING, M.J., BROWN, I.L., MACPHERSON, S.G., TURNER, M.A. & KAYE, S.B. (1989). Excision of residual masses after platinum based chemotherapy for non-seminomatous germ cell tumours. Eur. J. Cancer, 25, 1689-1694.

HENDRY, W.F., A'HERN, R.P., HETHERINGTON, J.W., PECKHAM, M.J., DEARNALEY, D.P. & HORWICH, A. (1993). Para-aortic lymphadenectomy after chemotherapy for metastatic non-seminomatous germ cell tumours: prognostic value and therapeutic benefit. *Br. J. Urol.*, **71**, 208–213.

JANSEN, R.L.H., SYLVESTER, R., SLEYFER, D.TH., TEN BOKKEL HUININK, W.W., KAYE, S.B., JONES, W.G., KEIZER, H.J., VAN OOSTEROM, A.T., MEYER, S., VENDRIK, C.P.J., DE PAUW, M. & STOTER, G. FOR THE EORTC GU GROUP (1991). Long-term follow-up of non-seminomatous testicular cancer patients with mature teratoma or carcinoma at post-chemotherapy surgery. *Eur. J. Cancer*, **27**, 695–698.

KULKARNI, R.P., REYNOLDS, K.W., NEWLANDS, E.S., DAWSON, P.M., MAKEY, A.R., THEODOROU, N.A., BRADLEY, J., BEGENT, R.H.J., RUSTIN, G.J.S. & BAGSHAWE, K.D. (1991). Cytoreductive surgery in disseminated non-seminomatous germ cell tumours of testis. *Br. J. Surg.*, **78**, 226–229.

LEVITT, M.D., REYNOLDS, P.M., SHEINER, H.J. & BYRNE, M.J. (1985). Non-seminomatous germ cell testicular tumours: residual masses after chemotherapy. *Br. J. Surg.*, **72**, 19–22.

LOGOTHETIS, C.J., SAMUELS, M.L., TRINDADE, A. & JOHNSON, D.E. (1982). The growing teratoma syndrome. *Cancer*, **50**, 1629–1635.

MANDELBAUM, I., YAW, P.B., EINHORN, L.H., WILLIAMS, S.D., ROWLAND, R.G. & DONOHUE, J.P. (1983). The importance of one-stage median sternotomy and retroperitoneal lymph node dissection in disseminated testicular cancer. *Ann. Thorac. Surg.*, **36**, 524–527.

MEAD, G.M., STENNING, S.P., PARKINSON, M.C., HORWICH, A., FOSSA, S.D., WILKINSON, P.M., KAYE, S.B., NEWLANDS, E.S. & COOK, P.A. FOR THE MEDICAL RESEARCH COUNCIL TESTICULAR TUMOUR WORKING PARTY (1992). The second medical research council study of prognostic factors in non-seminomatous germ cell tumors. *J. Clin. Oncol.*, **10**, 85–94.

MOSTOFI, F.K. & SOBIN, L.H. (1977). Histopathological typing of testis tumours. In *International Histological Classificatiion of Tumors*, No. 16 pp. 27–31. World Health Organization: Geneva.

PIZZOCARO, G., SALVIONI, R., PASI, M., ZANONI, F., MILANI, A., PILOTTI, S. & MONFARDINI, S. (1985). Early resection of residual tumor during cisplatin, vinblastine, bleomycin combination chemotherapy in stage III and bulky stage II nonseminomatous testicular cancer. *Cancer*, **56**, 249–255.

PUGH, R.C.B. (1976). Testicular tumours, introduction. In *Pathology of the Testis*, Pugh, R.C.B. (ed.) pp. 139–162. Blackwell Scientific Publications: Oxford.

QVIST, H., FOSSA, S.D., OUS, S., HOIE, J., STENWIG, A.E. & GIERCKSKY, K.E. (1991). Post-chemotherapy tumor residuals in patients with advanced nonseminomatous testicular cancer. Is it necessary to resect all residual masses? *J. Urol.*, **145**, 300–303.

ROTH, B.J., GREIST, A., KUBILIS, P.S., WILLIAMS, S.D. & EINHORN, L.H. (1988). Cisplatin-based combination chemotherapy for disseminated germ cell tumors: long-term follow-up. *J. Clin. Oncol.*, **8**, 1239–1247.

STAEHLER, G., WIESEL, M., CLEMM, C., GOKEL, J.M. & MARCHNER, M. (1989). Significance of salvage lymphadenectomy in the therapeutical concept of advanced nonseminomatous germ cell tumors. *Urol. Int.*, **44**, 84–86.

STEYERBERG, E.W., KEIZER, H.J., ZWARTENDIJK, J., VAN RIJK, G.L., VAN GROENINGEN, C.J., HABBEMA, J.D.F. & STOTER, G. (1993). Prognosis after resection of residual masses following chemotherapy for metastatic non-seminomatous testicular cancer: a multivariate analysis. *Br. J. Cancer*, **68**, 195–200.

TAIT, D., PECKHAM, M.J., HENDRY, W.F. & GOLDSTRAW, P. (1984). Post-chemotherapy surgery in advanced non-seminomatous germ-cell testicular tumours: the significance of histology with particular reference to differentiated (mature) teratoma. *Br. J. Cancer*, **50**, 601–609.

TIFFANY, P., MORSE, M.J., BOSL, G., VAUGHAN, E.D., SOGANI, P.C., HERR, H.W. & WHITMORE, W.F. (1986). Sequential excision of residual thoracic and retroperitoneal masses after chemotherapy for stage III germ cell tumors. *Cancer*, **57**, 978–983.

TONER, G.C., PANICEK, D.M., HEELAN, R.T., GELLER, N.L., LIN, S.-Y., BAJORIN, D., MOTZER, R.J., SCHER, H.I., HERR, H.W., MORSE, M.J, FAIR, W.R., SOGANI, P.C., WHITMORE, W.F., MCCORMACK, P.M., BAINS, M.S., MARTINI, N. & BOSL, G.J. (1990). Adjunctive surgery after chemotherapy for non-seminomatous germ cell tumors: recommendations for patient selection. *J. Clin. Oncol.*, **8**, 1983–1994.

WILLIAMS, S.D., BIRCH, R., EINHORN, L.H., IRWIN, L., GRECO, F.A. & LOEHRER, P.J. (1987). Treatment of disseminated germ-cell tumors with cisplatin, bleomycin, and either vinblastine or etoposide. *N. Engl. J. Med.*, **316**, 1435–1440.